# Supplementary material for: Snake venom NAD glycohydrolases: primary structures, genomic location, and gene structure
Source: PeerJ. 2019 Feb 6;7:e6154. doi: 10.7717/peerj.6154 (PMC6368836; doi:10.7717/peerj.6154)
Supplement: Supplemental Information 1 [file peerj-07-6154-s001.docx]

**Supplementary Information for**

**Snake venom NAD glycohydrolases: Primary structures, genomic location, and gene structure**

Ivan Koludarov^1^ and Steven D. Aird^1,2^

^1^Ecology and Evolution Unit and ^2^Division of Faculty Affairs, Okinawa Institute of Science and Technology, 1919-1 Tancha, Onna-son, Kunigami-gun, Okinawa, Japan 904-0495

The following are the venom gland transcriptomic sequences employed in this study.

>*Micrurus carvalhoi* NAD Glycohydrolase TRINITY_DN61384_c0_g1_i1|m.5640:

ATGCCCTTTCAAAACAGTTATTCCTGGACAAGGAAACAGAAACTGTTTTTGACGGGGGTGGTAGTGCTTTTGGGCACCATTACTGTTTTTGTGGTTTTTGGACTGCTCAAGCTTGGATGGAAGAAGAACCACGCTGCAGAAGAACAGCAGTGGAAAGGCAGAGGGACCACTGAACACCTCCTGGAAATTGTCCTGGGAAGATGTTACAACTTCATCAATACAATAAATCCTGAACTCAGAAATAAAGATTGTGTCAAAATACGGAAACTATTTGAACAGGCTTTTTTGTACAAGGACCCATGCAGTATAACCAAAGAAGATTACCAGCCTTTAATGGACCTGGCAAGATATCCCATACCATGCAACAAGTCCTTATTTTGGAGCAAAACATATGATCTGGTACATCATTACACGAAAACAAATCATAATTTCCTCACCTTGGAAGATACTTTGCTAGGCTACATAGCAGATGGGATTACTTGGTGTGGAAAGCCCTCTGATTCAGGAATCAATTATGAATCTTGTCCAAAATGGACTGAGTGTGAAAATAACCCCAATTCAGTATATTGGAAATTGGCATCTAAGATGTTTGCAGAAACATCCTGTGGAACAGTCCAAGTGATGCTCAATGGATCAATAGAGAATGGAGCTTTTAGACAAAGCAGCATTTTTGGCAGTGTTGAAATTGTTAACTTAAACCCAAAGAAAGTTTCCAAGATGCAGATCTGGCTAATGCATGATATTGGTGGACCCCAGAGGGAATCTTGCACAGGGGATTCCATTGCACAGTTGAGAGAGATCTTGGAAAACCGAAGTATAATTGTCTCCTGCGAAGACAATTACAGGCCGGCACAGTTACTTCAGTGTACTAGGAACCCCAACCACGCCACCTGCACAATTCGTTCCTGA

>*Micrurus corallinus* NAD Glycohydrolase TRINITY_DN100482_c0_g2_i2|m.92:

ATGCCCTTTCAAAACAGTTATTCCTGGACAAGGAAACAGAAACTGTTTTTGACGGGGGTGGTAGTGCTTTTGGGCACCATTACTGTTTTTGTGGTTTTTGGACTGCTCAAGCTTGGATGGAAGAAGAACCACGCTGCAGAAGAACAGCAGTGGAAAGGCAAAGGGACCACTGAACACCTCCTGGAAATTGTCCTGGGAAGATGTTACAACTTCATCAATACAATAAATCCTGAACTCAGAAATAAAGATTGTGTCAGAATACAGAAACTATTTAAACAGGCTTTTCTGTACAAGGACCCATGCAGTATAACCAAAGAAGATTACCAGCCTTTAATGGACCTGGCAAGATATTCCATACCATGCAACAAGTCCTTATTTTGGAGCAAAACATCTGATCTGGCACATCATTACACGAAAACAAATCATAATTTCCTCACCTTGGAAGATACTTTGCTAGGCTACATAGCAGATGGGATTACTTGGTGTGGAAAGCCCTCTGATTCAGGAATCAATTATGAATCTTGTCCAAAATGGACTGAGTGTGAAAATAACCCCAGTTCAGTATATTGGAAATTGGCATCTAAGATGTTTGCAGAAACAGCCTGTGGAACAGTCCAAGTGATGCTCAATGGATCAATAGAGAATGGAGCTTTTAGACAAAGCAGCATTTTTGGCAGTGTTGAAATTGTGAACTTAAACCCAGAGAAAGTTTCCAAGATGCAGATCTGGCTAATGCATGATATTGGTGGACACCAGTGGGAATCTTGCACAGGGCCTTCCATTGCACAGTTGAGAGAGATCTTGGAAAACCGAAGTATAATTGTCTCCTGCGAAGACAATTACAGGCCGGCACAGTTACTTCAGTGTACTAGGAACCCCAACCACACCACCTGCACAATTCGTTCCTGA

>*Micrurus lemniscatus* NAD Glycohydrolase TRINITY_DN22889_c0_g1_i1|m.65259:

ATGCCCTTTCAAAACAGTTATTCCTGGACAAGGAAACAGAAACTGTTTTTGACGGGGGTGGTAGTGCTTTTGGGCACCATTACTGTTTTTGTGGTTTTTGGACTGCTCAAGCTTGGATGGAAGAAGAACCACGCTGCAGAAGAACAGCAGTGGAAAGGCAGAGGGACCACTGAACACCTCCTGGAAATTGTCCTGGGAAGATGTTACAACTTCATCAATACAATAAATCCTGAACTCAGAAATAAAGATTGTGTCAAAATACGGAAACTATTTGAACAGGCTTTTTTGTACAAGGACCCATGCAGTATAACCAAAGAAGATTACCAGCCTTTAATGGACCTGGCAAGATATTCCATACCATGCAACAAGTCCTTATTTTGGAGCAAAACATATGATCTGGTACATCATTACACGAAAACAAATCATAATTTCCTCACCTTGGAAGATACTTTGCTAGGCTACATAGCAGATGGGATTACTTGGTGTGGAAAGCCCTCTGATTCAGGAATCAATTATGAATCTTGTCCAAAATGGACTGAGTGTGAAAATAACCCCAATTCAGTATATTGGAAATTGGCATCTAAGATGTTTGCAGAAACATCCTGTGGAACAGTCCAAGTGATGCTCAATGGATCAATAGAGAATGGAGCTTTTAGACAAAGCAGCATTTTTGGCAGTGTTGAAATTGTTAACTTAAACCCAGAGAAAGTTTCCAAGATGCAGATCTGGCTAATGCATGATATTGGTGGACCCCAGAGGGAATCTTGCACAGGGGATTCCATTGCACAGTTGAGAGAGATCTTGGAAAACCGAAGTATAATTGTCTCCTGCGAAGACAATTACAGGCCGGCACAGTTACTTCAGTGTACTAGGAACCCCAACCACGCCACCTGCACAATTCGTTCCTGA

>*Micrurus paraensis* NAD Glycohydrolase TRINITY_DN86064_c0_g1_i1|m.15110:

TTTCACACAGGAAAGCAGTGGTATCAACGCAGAGTACATGGGGAAGGCAGAAAGTGCAGCTGTTTCTACTTTTCAGAATCTTCTACTAAGATGCCCTTTCAAAACAGTTATTCCTGGACAAGGAAACAGAAACTGTTTTTGACGGGGGTGGTAGTGCTTTTGGGCACCATTACTGTTTTTGTGGTTTTTGGACTGCTCAAGCTTGGATGGAAGAAGAACCACGCTGCAGAAGAACAGCAGTGGAAAGGCAAAGGGACCACTGAACACCTCCTGGAAATTGTCCTGGGAAGATGTTACAACTTCATCAATACAATAAATCCTGAACTCAGAAATAAAGATTGTGTCAGAATACAGAAACTATTTAAACAGGCTTTTCTGTACAAGGACCCATGCAGTATAACCAAAGAAGATTACCAGCCTTTAATGGACCTGGCAAGATATTCCATACCATGCAACAAGTCCTTATTTTGGAGCAAAACATCTGATCTGGCACATCATTACACGAAAACAAATCATAATTTCCTCACCTTGGAAGATACTTTGCTAGGCTACATAGCAGATGGGATTACTTGGTGTGGAAAGCCCTCTGATTCAGGAATCAATTATGAATCTTGTCCAAAATGGACTGAGTGTGAAAATAACCCCAGTTCAGTATATTGGAAATTGGCATCTAAGATGTTTGCAGAAACAGCCTGTGGAACAGTCCAAGTGATGCTCAATGGATCAATAGAGAATGGAGCTTTTAGACAAAGCAGCATTTTTGGCAGTGTTGAAATTGTGAACTTAAACCCAGAGAAAGTTTCCAAGATGCAGATCTGGCTAATGCATGATATTGGTGGACACCAGTGGGAATCTTGCACAGGGCCTTCCATTGCACAGTTGAGAGAGATCTTGGAAAACCGAAGTATAATTGTCTCCTGCGAAGACAATTACAGGCCGGCACAGTTACTTCAGTGTACTAGGAACCCCAACCACGCCACCTGCACAATTCGTTCCTGA

>*Micrurus spixii* NAD Glycohydrolase TRINITY_DN121140_c2_g1_i1|m.22327:

ATGCCCTTTCAAAACAGTTATTCCTGGACAAGGAAACAGAAACTGTTTTTGACGGGGGTGGTAGTGCTTTTGGGCACCATTACTGTTTTTGTGGTTTTTGGACTGCTCAAGCTTGGATGGAAGAAGAACCACGCTGCAGAAGAACAGCAGTGGAAAGGCAAAGGGACCACTGAACACCTCCTGGAAATTGTCCTGGGAAGATGTTACAACTTCATCAATACAATAAATCCTGAACTCAGAAATAAAGATTGTGTCAGAATACAGAAACTATTTAAACAGGCTTTTCTGTACAAGGACCCATGCAGTATAACCAAAGAAGATTACCAGCCTTTAATGGACCTGGCAAGATATTCCATACCATGCAACAAGTCCTTATTTTGGAGCAAAACATCTGATCTGGCACATCATTACACGAAAACAAATCATAATTTCCTCACCTTGGAAGATACTTTGCTAGGCTACATAGCAGATGGGATTACTTGGTGTGGAAAGCCCTCTGATTCAGGAATCAATTATGAATCTTGTCCAAAATGGACTGAGTGTGAAAATAACCCCAGTTCAGTATATTGGAAATTGGCATCTAAGATGTTTGCAGAAACAGCCTGTGGAACAGTCCAAGTGATGCTCAATGGATCAATAGAGAATGGAGCTTTTAGACAAAGCAGCATTTTTGGCAGTGTTGAAATTGTGAACTTAAACCCAGAGAAAGTTTCCAAGATGCAGATCTGGCTAATGCATGATATTGGTGGACACCAGTGGGAATCTTGCACAGGGCCTTCCATTGCACAGTTGAGAGAGATCTTGGAAAACCGAAGTATAATTGTCTCCTGCGAAGACAATTACAGGCCGGCACAGTTACTTCAGTGTACTAGGAACCCCAACCACACCACCTGCACAATTCGTTCCTGA

>*Micrurus surinamensis* NAD Glycohydrolase TRINITY_DN77054_c0_g1_i1|m.2918:

TTTTCCCAGTCACGACAATTGCAGTGGTATCAACGCAGAGTACATGGGGAAGGCAGAAAGTGCAGCTGTTTCTACTTTTCAGAATCTTCTACTAAGATGCCCTTTCAAAACAGTTATTCCTGGACAAGGAAACAGAAACTGTTTTTGACGGGGGTGGTAGTGCTTTTGGGCACCATTACTGTTTTTGTGGTTTTTGGACTGCTCAAGCTTGGATGGAAGAAGAACCACGCTGCAGAAGAACAGCAGTGGAAAGGCAAAGGGACCACTGAACACCTCCTGGAAATTGTCCTGGGAAGATGTTACAACTTCATCAATACAATAAATCCTGAACTCAGAAATAAAGATTGTGTCAAAATACGGAAACTATTTGAACAGGCTTTTTTGTACAAGGACCCATGCAGTATAACCAAAGAAGATTACCAGCCTTTAATGGACCTGGCAAGATATTCCATACCATGCAACAAGTCCTTATTTTGGAGCAAAACATCTGATCTGGCACATCATTACACGAAAACAAATCATAATTTCCTCACCTTGGAAGATACTTTGCTAGGCTACATAGCAGATGGGATTACTTGGTGTGGAAAGCCCTCTGATTCAGGAATCAATTATGAATCTTGTCCAAAATGGACTGAGTGTGAAAATAACCCCAGTTCAGTATATTGGAAATTGGCATCTAAGATGTTTGCAGAAACAGCCTGTGGAACAGTCCAAGTGATGCTCAATGGATCAATAGAGAATGGAGCTTTTAGACAAAGCAGCATTTTTGGCAGTGTTGAAATTGTGAACTTAAACCCAGAGAAAGTTTCCAAGATGCAGATCTGGCTAATGCATGATATTGGTGGACACCAGTGGGAATCTTGCACAGGGCCTTCCATTGCACAGTTGAGAGAGATCTTGGAAAACCGAAGTATAATTGTCTCCTGCGAAGACAATTACAGGCCGGCACAGTTACTTCAGTGTACTAGGAACCCCAACCACGCCACCTGCACAATTCGTTCCTGA

>*Ovophis okinavensis* NAD Glycohydrolase comp19518_c0_seq1 (reversed): (Partial)

AGAGTGGAAAGGCAGAGGAACCACTAAACACCTGCTGGAAATTGTCCTGGGAAGATGTTACAACTTCATTAATACAATAAATCCTGAACTTAGAAATAAAGATTGTCTCAAAGTATGGAAACTATTTGAACAGGCTTTTCTGTACAAGGATCCGTGCAGAGTGACCGAAGAAGATTACCAGCCTTTAATGGACCTGGCAAGATATTCCATACCATGCAACAAGTCCTTATTCTGGAGCAAAACATATGACCTGGCACATCATTACACGAAAACCAATAATGATTTCCTCACCTTGGAAGATACTTTGCTAGGCTACATAGCAGATGGGATTTCCTGGTGTGGAAATCCCTCCAATTCAGGAGTCAATTATGAATCTTGTCCAAAATGGACTGAGTGTGAAAATAATCCCAGTTCAGTATATTGGAAATTGGCATCTAAGATGTTTGCAGAAACATCCTGTGGAACAGTTCAAGTGATGCTCAATGGATCAACAATGTCTGGAGCATTTAGAAAAAGCAGCATTTTTGGCAGTGTTGAAATAGTTAACTTAAACCCAAAGGAAGTTTCCAAGATGCAGATTTGGTTA

>*Protobothrops elegans* NAD Glycohydrolase comp350_c0_seq1:

CAACGCAGAGTACATGGGGGAGGCAGAAAGTGCAGCTGCTGCTGCTCTTCAAAAACTTCTACTAAGATGCCCTTTCAAAACAGTTCTTCCTGGACAAAGAAACAGAAACTGATTTTGACAGGGGTGATAGTGGTGCTTTTGGGCACCTTGACTGTTTTTGTGGTTTTTGGACTGCTCAGGCTTGGAAGGAAGAAGATCCCCATTGCAGAAGAACAGGAGTGGAAAGGCAGAGGAACCACTGAACACCTGCTGGAAATTGTCCTGGGAAGATGTTACAACTTCATTAATACAATAAATCCTGAACTTAGAAATAAAGATTGTCTCAAAGTATGGAAACTATTTGAACAGGCTTTTCTGTACAAGGATCCGTGCAGAGTGGCCAAAGAAGATTACCAGCCTTTAATGGACCTGGCAAGATATTCCATACCATGCAACAAGTCCTTATTCTGGAGCAAAACATATGACCTGGCACATCATTACACGAAAACCAATAATGATTTCCTCACCTTGGAAGATACTTTGCTAGGCTACATAGCAGATGGGATTTCCTGGTGTGGAAATCCCTCCAATTCAGGAGTCAATTATGAATCTTGTCCAAAATGGACTGAGTGTGAAAATAATCCCAGTTCAGTATATTGGAAATTGGCATCTAAGATGTTTGCAGAAACATCCTGTGGAACAGTTCAAGTGATGCTCAATGGATCAACAATGTCTGGAGCATTTAGAAAAAGCAGCATTTTTGGCAGTGTTGAAATAGTTAACTTAAACCCAAAGGAAGTTTCCAAGATGCAGATTTGGTTAATGCATGACATTGATGGACCTCAGAGGGAATCTTGCACAGGACATTCCATTGCACAGTTGAGAGAGATCTTGGAAAACAGAAATATAAGTGTCTCCTGCGAAGACAATTACAGGCCAGCACAGTTACTTCAGTGTACTAGAAACCCCAACCATACTGCCTGCAAAGTTTGTTCCTGAAATCATGGGGACGACAGAAGAGTGAATGTCATCAGATTCAGAGAAATTCAACAGATAATATCATTGTTTAATTTAGAGAGCTTCTTTCTATCACATAATTTTATATCACTCAATACGTTAAACGTTTCTGCTTTTCTAAGTGTCAATGTTCGTTATATTTTATTGTGTGTGTCCTTGTTTGCTGCTGCTAATAAAGGTAACAAAATATTTTAATAAAATATCCATTTATATTTTCTTCATTTTCTCTGACTCCCATTAAGTGCCAAGCAAGACAATCGAGAATGGTTAAGTATTTGTTACAGGCTAAGTGGGAATTTGAACATAGGTCTTTCTAGCTCTTGGCAAGCCATTGTCCTCTCTGTTTCAACTCTAAATTAAAACTGAAGGTCTGAAAAAGCCCTTGCATTTCTTAGTGATAGAATAGAATAAAATAGAGCTGGAAGGGACCTTGGA

>*Protobothrops flavoviridis* NAD Glycohydrolase comp3789_c0_seq1 (reversed): (Partial)

CGCAGAGTACATGGGGGAGGCAGAAAGTGCAGCTGCTGCTGCTCTTCAAAAACTTCTACTAAGATGCCCTTTCAAAACAGTTCTTCCTGGACAAAGAAACAGAAACTGATTTTGACAGGGGTGATAGTGGTGCTTTTGGGCACCTTGACTGTTTTTGTGGTTTTTGGACTGCTCAGGCTTGGAAGGAAGAAGATCCCCATTGCAGAAGAACAGGAGTGGAAAGGCAGAGGAACCACTGAACACCTGCTGGAAATTGTCCTGGGAAGATGTTACAACTTCATTAATACAATAAATCCTGAACTTAGAAATAAAGATTGTCTCAAAGTATGGAAACTATTTGAACAGGCTTTTCTGTACAAGGATCCGTGCAGAGTGGCCGAAGAAGATTACCAGCCTTTAATGGACCTGGCAAGATATTCCATACCATGCAACAAGTCCTTATTCTGGAGCAAAACATATGACCTGGCACATCATTACACGAAAACCAATAATGATTTCCTCACCTTGGAAGATACTTTGCTAGGCTACATAGCAGATGGGATTTCCTGGTGTGGAAATCCCTCCAATTCAGGAGTCAATTATGAATCTTGTCCAAAATGGACTGAGTGTGAAAATAATCCCAGTTCAGTATATTGGAAATTGGCATCTAAGATGTTTGCAGAAACATCCTGTGGAACAGTTCAAGTGATGCTCAATGGATCAACAATGTCTGGAGCATTTAGAAAAAGCAGGTATTCTCTTTCCATTCATGTGCTTAAAATGCTTCTAAAGTAAACAAGGAAAGTTTTCTTCACCATTTATTTGTCGATATTTCTGACCAAGCATCTCTATGTCAGTCATGGGTATCTGCAAGATATGGAGTAAAGAAGGTAGTCACAATGATGTGATTGAAGTACTACTGTAGGAGATGATCAGGCTGAGACTGAGGAGGGGTCAAAGGCATCATCAATTCTGAATCCCTTCACGCCCACAAGATGGATCTTGAAATGCATCTGAACTAAGTCCAGCCCACCTTGAATTCCTTATCTACCACCAAATTGCTTGACTATTAGTAGTTCCGATTCTTGTAGAGAGCTTAAGCT
